# Paraganglioma Presenting as Postpartum Fever of Unknown Origin

**DOI:** 10.1155/2015/864719

**Published:** 2015-07-07

**Authors:** Shraddha Narechania, Amrita Bath, Laleh Ghassemi, Chetan Lokhande, Abdo Haddad, Ali Mir Yousuf, Jessica Marquard, K. V. Gopalakrishna

**Affiliations:** ^1^Internal Medicine Residency, Fairview Hospital, Cleveland, OH 44111, USA; ^2^Anesthesia Department, Fairview Hospital, Cleveland, OH 44111, USA; ^3^Cleveland Clinic Taussig Cancer Institute, Fairview Hospital, Cleveland, OH 44111, USA; ^4^Strongsville Family Health and Surgery Center, 16761 Southpark Center, Strongsville, OH 44136, USA; ^5^Genomic Medicine Institute, 9500 Euclid Avenue NE5, Cleveland, OH 44195, USA

## Abstract

A young healthy postpartum mother presented with intermittent high fevers and tachycardia. Appropriate testing was done to rule out infectious causes including pan cultures but no identifiable infectious source was found. A CT of the abdomen showed a retroperitoneal mass with two small pulmonary nodules and a bony metastatic lesion. She was found to have stage 4 extra-adrenal paraganglioma with metastases to the lungs and spine. She underwent resection of the mass and is currently undergoing palliative radiation to the spine for pain control. Subsequent genetic testing identified a likely pathogenic variant in *SDHB*, confirming a diagnosis of Hereditary Paraganglioma-Pheochromocytoma syndrome.

## 1. Introduction

We report a rare and unique case of a young primigravida female patient who developed postpartum fever. After appropriate diagnostic testing, the patient was found to have a retroperitoneal paraganglioma. We report this case to emphasize that pheochromocytoma or paraganglioma should be considered in the differential diagnosis of postpartum fever especially with a history of preeclampsia during pregnancy.

## 2. Case Presentation

**Figure 1:**
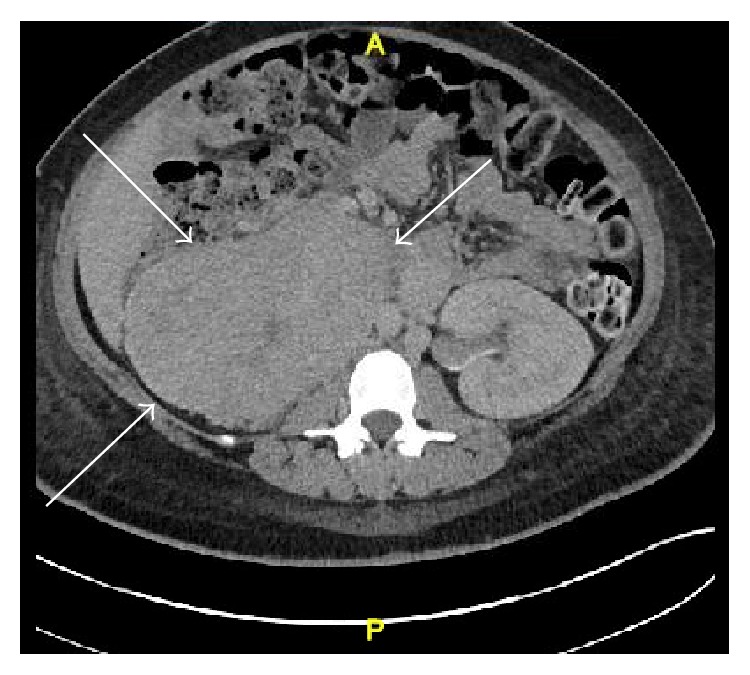
CT scan abdomen/pelvis with contrast showing large retroperitoneal mass.

**Figure 2:**
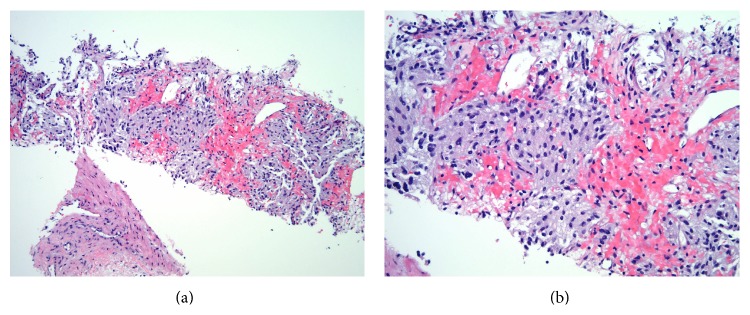
Microscopic examination of renal biopsy specimen in 100x view (a) and 200x view (b) showing cellular epithelioid proliferation arranged in nests and composed of round to ovoid cells with hyperchromatic nuclei, inconspicuous nucleoli, and granular amphophilic cytoplasm.

**Figure 3:**
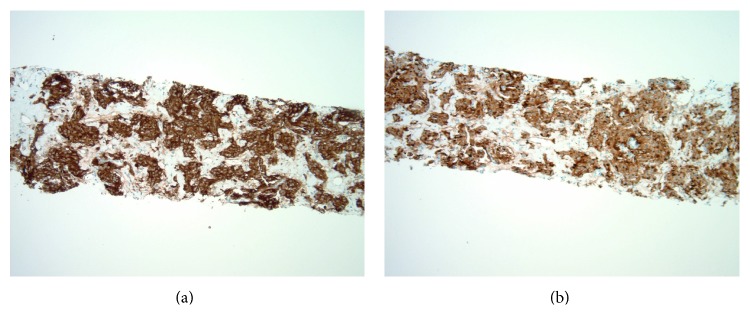
CT guided renal mass biopsy showing positive staining for synaptophysin (a) and chromogranin (b).

A 21-year-old primigravida at 37-week gestation presented to the outpatient clinic for a routine obstetrical examination. Her pregnancy course had been uneventful. Her blood pressure at this visit was 184/108. She was asymptomatic except for occasional mild headaches. A urine dipstick showed 3+ proteinuria. Patient was diagnosed with preeclampsia and was admitted to the hospital for emergency induction of labor. She was started on IV magnesium for preeclampsia and labor was induced with oxytocin. She underwent an emergency cesarean section due to fetal decelerations during induction. Subsequently, she delivered a healthy male infant by low transverse C section. Four days after partum, she started spiking high fevers up to 39.5 degrees Celsius and developed tachycardia with heart rate of 140 to 150/min. She was otherwise completely asymptomatic and clinical exam was negative for any localizing signs. Her cesarean section incision looked clean; she had no breast tenderness. Infectious work-up including blood and urine cultures was negative. Her chest X-ray was normal; EKG showed sinus tachycardia. She was empirically started on broad spectrum antibiotics. After 48 hours of IV antibiotics, patient continued to spike high fevers and was still tachycardic. The differential diagnosis of endometritis and septic thrombophlebitis was high on the list even though her abdominal exam was benign. Computerized tomography (CT) scan of the abdomen and pelvis with contrast (Figure 1) was ordered to rule out septic thrombophlebitis, which showed a large right retroperitoneal 9.2 cm anteroposterior × 14 cm transverse × 12.5 cm craniocaudal mass with severe right renal hydronephrosis. Due to absence of any fat layer between the kidney and the mass, it was felt to be coming from the lower pole of the right kidney. The mass extended superiorly to the inferior vena cava (IVC) with a possible tumor thrombus in the IVC. The liver was mildly enlarged measuring 19.6 cm with fatty infiltration. The first segment of the sacrum showed a 3 cm × 2.1 cm × 2.6 cm lytic lesion. CT scan of the chest showed 2 pulmonary nodules measuring 7 mm and 11 mm in the left and right lower lobes of the lung, respectively. Due to the above imaging findings, suspicion was high for a stage 4 metastatic cancer arising from the kidney. Renal ultrasound confirmed a large retroperitoneal mass with hydronephrosis. MRI lumbar spine reported an abnormal marrow signal in the S1–S3 segments and an exophytic bony metastatic lesion in the dorsal aspect of S1 causing severe narrowing of the thecal sac. MRI abdomen/pelvis confirmed extension of the retroperitoneal mass into the inferior vena cava (IVC). MRI brain and CT brain were obtained showing calvarial lesions consistent with hemangiomas. A percutaneous CT guided right renal mass biopsy (Figure 2) revealed a cellular epithelioid proliferation arranged in nests and composed of round to ovoid cells with hyperchromatic nuclei, inconspicuous nucleoli, and granular amphophilic cytoplasm. Immunohistochemical staining was negative for HMB-45, S100, PAX-8, cytokeratin AE1/AE3, CD117, and DOG-1 but was positive for synaptophysin and chromogranin (Figure 3). The overall morphological and immunohistochemical findings were consistent with a diagnosis of paraganglioma. 24-hour urine testing revealed high levels of urinary nor epinephrine which was 862 mcg/24 hours (normal range 15 to 100 mcg/24 hours) and dopamine which was 902 mcg/24 hours (normal range 52 to 480 mcg/24 hours). Plasma-free metanephrine levels were also elevated at 1690 pg/mL (normal range 18 to 101 pg/mL). The patient continued to remain severely hypertensive after delivery and was started on combined alpha and beta blockade with doxazosin 8 mg daily and propranolol 40 mg twice daily. The patient underwent right sided nephrectomy, removal of the retroperitoneal mass, and resection of the perirenal IVC. Her postoperative course was complicated by severe hypotension and extensive bilateral lower extremity deep venous thromboses in the external iliac and common femoral veins. She required vasopressors for two days and was started on therapeutic anticoagulation with enoxaparin for the DVTs. She was discharged home 2 weeks after her surgery. A metaiodobenzylguanidine (MIBG) whole body scan done later as an outpatient did not demonstrate any focal abnormal uptake. Whole body PET scan showed increased FDG uptake in 3 pulmonary nodules, a large lytic sacral lesion, and a T9 vertebral body lesion all consistent with metastases. The patient underwent gamma knife palliative radiation of the S1 lesion and was started on Zometa for her bony metastases. The tyrosine kinase receptor inhibitor Sunitinib was started to treat her systemic disease.

The patient underwent genetic counseling and pursued genetic testing for multiple hereditary paraganglioma syndromes. She had no known family history of paraganglioma, pheochromocytoma, kidney cancer, or thyroid cancer. Next generation sequencing of* MAX*,* NF1*,* RET*,* SDHA*,* SDHAF2*,* SDHB*,* SDHC*,* SDHD*,* TMEM127*, and* VHL* identified a likely pathogenic variant in* SDHB*, c.418G>T (p.Val140Phe). This result confirmed a diagnosis of Hereditary Paraganglioma-Pheochromocytoma syndrome, an autosomal dominant condition with reduced penetrance.

## 3. Discussion

Paragangliomas, also sometimes called extra-adrenal pheochromocytomas, are rare neuroendocrine tumors arising from sympathetic and parasympathetic paraganglia outside the adrenal medulla. They can occur at almost any site in the body including head, neck, thorax, and abdominal cavity. Most of the thoracic and abdominal paragangliomas arise from sympathetic ganglia and are secretory while head and neck paragangliomas are generally nonsecretory as they arise from parasympathetic ganglia [1, 2]. They are closely related to pheochromocytomas and are clinically undifferentiable from them causing symptoms attributable to excessive catecholamine secretion such as episodic hypertension, diaphoresis, headaches, and palpitations. Histologically, the main difference between these is that paragangliomas arise from extra-adrenal chromaffin tissue cells whereas pheochromocytomas arise from adrenal chromaffin tissue [3].

Most paragangliomas occur sporadically; however, up to 30% are due to a hereditary predisposition syndrome [4]. All individuals diagnosed with a paraganglioma or pheochromocytoma should be referred for genetic counseling. Factors that increase the likelihood of a hereditary cause include early age at diagnosis, extra-adrenal location, and positive family history.* SDHB-*associated paragangliomas are malignant in up to 37% of cases [5]. Individuals with Hereditary Paraganglioma-Pheochromocytoma syndrome should be followed with annual biochemical screening for functional paraganglioma and periodic imaging of the neck, chest, abdomen, and pelvis. Their first-degree relatives have a 50% chance of having the familial pathogenic variant and should have genetic counseling and testing.

Paragangliomas are seen most commonly located in the inferior para-aortic region; however, they also occur in other regions like the urinary bladder, mediastinum, head, and neck [6]. Paragangliomas rarely produce epinephrine, majority of them producing norepinephrine or a combination of norepinephrine and dopamine and some rarely even producing dopamine alone. Most adrenal tumors on the other hand produce epinephrine and norepinephrine [7]. Majority of these tumours are benign and only up to 3% are malignant [8].

The occurrence of paragangliomas in pregnancy is extremely rare and has been reported to be about 5 per million pregnancies in some studies to 18.5 per million live births in others [6, 9, 10]. Paragangliomas have been reported to occur at various sites during pregnancy including bladder and heart [11, 12]. We report a rare and unique case of a paraganglioma presenting as postpartum fever and hypertension in a young female. Kleiner et al. had, in 1982, described a similar case of paraganglioma presenting as postpartum fever [13]. To the best of our knowledge, this is the first reported case of a paraganglioma causing IVC thrombosis with extensive lower extremity deep venous thrombosis and the second reported case of paraganglioma presenting as postpartum fever.

Timely diagnosis and treatment are of paramount importance as the maternal and fetal mortality can be as high as 50% if this condition is undiagnosed and untreated [6]. A hypertensive crisis can be precipitated by uterine contractions during labor and vaginal delivery [10]. Data indicates that about 20% of patients pass through pregnancy undiagnosed due to the nonspecific nature of signs and symptoms and the much higher prevalence of gestational hypertension [14]. The best initial screening test recommended for diagnosis is the measurement of plasma-free metanephrines [15]. However, a general consensus has not been reached in regard to the diagnostic testing of pheochromocytoma or paraganglioma. It has been suggested that, for patients with a low pretest probability, testing with 24-hour urinary metanephrines and catecholamines is preferable as it can avoid high false positive rates seen with measurement of plasma-free metanephrines [16].

After biochemical confirmation, imaging is used to identify the location of the tumour. In pregnant females, the imaging modality of choice is Magnetic Resonance Imaging whose sensitivity can be as high as 90% in diagnosing paragangliomas and it minimizes fetal exposure to ionizing radiation [10, 14]. Ultrasound can also be used for detection but it is not the preferred modality as it is not as sensitive as MRI in detecting adrenal tumors [13]. The accuracy of ultrasound depends on the technical skill of the operator, the ultrasound quality, and the body habitus of the patient [17]. In a pregnant patient, the bulky uterus might preclude the detection of adrenal and retroperitoneal structures as might have happened in our case [13, 18].

Surgical resection is the mainstay of treatment during pregnancy [10]. Presurgical preparation of patients is done by initiating alpha blocking agents like phenoxybenzamine or doxazosin followed by beta blocking drugs like propranolol or atenolol to avoid unopposed alpha stimulation which can lead to a hypertensive crisis. In pregnant women the optimum time for removal of the tumor is either before 24 weeks if diagnosed that early or after delivery if diagnosed after 24 weeks. The second trimester is the safest for surgery due to the high risk of spontaneous abortion in the first trimester. Prolonged follow-up is necessary as these tumors have a high rate of recurrence.

## 4. Conclusion

Due to the extremely high mortality rate of this rare but treatable condition, it is essential to conduct a meticulous history taking and clinical examination so that early diagnosis and treatment can save maternal and fetal lives and improve clinical outcomes.
